# Supplementary material for: Medical Radiation Safety and COVID-19 Knowledge and Awareness among UAE Residents: A Cross-Sectional Study
Source: Healthcare (Basel). 2022 Jun 23;10(7):1174. doi: 10.3390/healthcare10071174 (PMC9317987; doi:10.3390/healthcare10071174)
Supplement: Supplementary file 1 [file healthcare-10-01174-s001.zip › healthcare-1760918-supplementary.pdf]

Demographic information section قسم المعلومات الشخصية

1. Gender نوع الجنس *

Mark only one oval.

- ( ) Male ذكر
- ( ) Female انثى

2. Age العمر *

____

3. Nationality الجنسية *

Mark only one oval.

- UAE National مواطن
- Expatriate مقيم

4. Marital status الحالة الاجتماعية *

Mark only one oval.

- Married متزوج
- Single اعزب

5. Study level المستوي الاكاديمي *

Mark only one oval.

- School مدرسي
- Diploma دبلوم
- Bachelor بكالريوس
- Master ماجستير
- Doctorate دكتوراه

6. Job title المجال الوظيفي *

Mark only one oval.

- Medical related job مجال طبي
- Non Medical related job مجال غير طبي
- Housewife ربة منزل
- No job لا اعمل

7. Work experience (years) if applicable عدد سنوات الخبرة العملية ان وجدت

____

8. Work Sector type قطاع العمل *

Mark only one oval.

- ( ) Public حكومي
- ( ) Private خاص
- ( ) Other: _______________

9. Address العنوان *

Mark only one oval.

- ( ) Abu Dhabi أبوظبي
- ( ) Al Ain العين
- ( ) Ajman عجمان
- ( ) Dubai دبي
- ( ) Fujairah الفجيرة
- ( ) Ras Al Khaimah رأس الخيمة
- ( ) Sharjah الشارقة
- ( ) Umm Al Quwain أم القيوين
- ( ) Other: _______________

COVID 19 Knowledge & Awareness section قسم المعرفة والوعي بفيروس كورونا

10. Have you received information about COVID19 safety procedures? هل تلقيت معلومات حول إجراءات السلامة الخاصة بـفيروس كورونا *

Mark only one oval.

- Yes نعم
- No لا

11. COVID 19 belongs to: الي اي عائلة ينتمي فيروس كورونا *

Mark only one oval.

- Closteroviridae family
- Circoviridae family
- Coronaviridae family
- I don't know لا أعلم

12. COVID19 was first reported in :اول حالة مكتشفة للفيروس كانت في *

Mark only one oval.

- USA أمريكيا
- CHINA الصين
- UK بريطانيا
- I don't know لا أعلم

13. COVID 19 most common symptom is: أكثر الأعراض شيوعًا لفيروس كورونا هي *

Mark only one oval.

- Dry cough سعال جاف
- Conjunctivitis احمرار العين) التهاب العين)
- Loss of speech فقدان الكلام
- I don't know لا أعلم

14. The risk factors of COVID 19 may include: قد تشمل عوامل خطر الإصابة بفيروس كورونا ما يلي: *

Mark only one oval.

- Asthma الربو
- Close contact with others التقارب الاجتماعي
- All of the above كل ما ذكر علاه
- I don't know لا أعلم

15. COVID19 prevention way may include: قد تشمل طريقة الوقاية من كورونا *

Mark only one oval.

- Stay home الجلوس بالمنزل
- Wash hands غسل اليدين باستمرار
- All of the above كل ما ذكر اعلاه
- I don't know لا أعلم

16. The most common used tool for COVID19 diagnosis is: الفحص الأكثر استخدامًا لتشخيص فيروس كورونا هي: *

Mark only one oval.

- CT scan الأشعة المقطعية
- PCR مسحة الانف و تحليل وجود الاجزاء الجينية للفيروس
- Chest X-ray تصوير الصدر بالاشعة العادية
- I don't know لا أعلم

Radiation safety Awareness section قسم الوعي بالسلامة من الاشعاع

17. If you have visited the radiology department, please select the imaging type from the below list. If not, please select NA إذا قمت بزيارة قسم الأشعة ، يرجى تحديد نوع التصوير من القائمة أدناه. إذا لم تقم بعمل فحص، يرجى تحديد لا يوجد *

Check all that apply.

- [ ] General X-ray الأشعة السينية العامة
- [ ] CT scan الاشعة المقطعية
- [ ] MRI التصوير بالرنين المغناطيسي
- [ ] Ultrasound الموجات فوق الصوتية
- [ ] DEXA scan قياس كثافة العظام بالاشعة
- [ ] Nuclear Medicine الطب النووي
- [ ] Dental imaging تصوير الأسنان
- [ ] Mammography تصوير الثدي الشعاعي
- [ ] Cath lab عمليات القسطرة
- [ ] Fluoroscopy التصوير الاشعاعي باستخدام الصبغة الملونة
- [ ] Portable/Mobile التصوير الاشعاعي المتنقل للمرضي بالعنابر و العناية المركزة
- [ ] NA لا يوجد

Other: [ ] ____________________

18. Have you received information about radiation safety? هل تلقيت معلومات حول الحماية من الإشعاع؟ *

Mark only one oval.

( ) Yes نعم

( ) No لا

Please rate your awareness level for the below items: يرجى تقييم مستوى وعيك للعناصر التالية

19. Ionizing radiation exposure can induce cancer يمكن أن يؤدي التعرض للإشعاع المؤين إلى الإصابة بالسرطان *

Mark only one oval.

- Not at all aware لا علم لي على الإطلاق
- Slightly aware أدرك قليلاً
- Somewhat aware أدرك إلى حد ما
- Moderately aware أدرك معلومات كافية
- Extremely aware أدرك معلومات كثيرة

20. X-ray is a harmful form of radiation الأشعة السينية هي شكل ضار من أشكال الإشعاع *

Mark only one oval.

- Not at all aware لا علم لي على الإطلاق
- Slightly aware أدرك قليلاً
- Somewhat aware أدرك إلى حد ما
- Moderately aware أدرك معلومات كافية
- Extremely aware أدرك معلومات كثيرة

21. Ultrasound imaging uses a safe form of radiation الموجات فوق الصوتية هي شكل آمن من أشكال الإشعاع *

Mark only one oval.

- Not at all aware لا علم لي على الإطلاق
- Slightly aware أدرك قليلاً
- Somewhat aware أدرك إلى حد ما
- Moderately aware أدرك معلومات كافية
- Extremely aware أدرك معلومات كثيرة

22. MRI uses a safe form of radiation التصوير بالرنين المغناطيسي هو شكل آمن من أشكال الإشعاع *

Mark only one oval.

- Not at all aware لا علم لي على الإطلاق
- Slightly aware أدرك قليلاً
- Somewhat aware أدرك إلى حد ما
- Moderately aware أدرك معلومات كافية
- Extremely aware أدرك معلومات كثيرة
